# Integrating logic-based machine learning and virtual screening to discover new drugs

**DOI:** 10.1186/1758-2946-4-S1-O10

**Published:** 2012-05-01

**Authors:** Christopher R Reynolds, Michael JE Sternberg

**Affiliations:** 1 Imperial College, London, N19 3LA, UK

## 

**I**nvestigational **N**ovel **D**rug **D**iscovery by **Ex**ample (INDDEx™) is a technology developed to guide hit to lead discovery by learning rules from existing active compounds that link activity to chemical substructure. INDDEx is based on Inductive Logic Programming [1], which learns easily interpretable *qualitative* logic rules from active ligands that give an insight into chemistry, relate molecular substructure to activity, and can be used to guide the next steps of drug design chemistry. Support Vector Machines weight the rules to produce a *quantitative* model of structure-activity relationships. Whereas earlier testing [2,3] was performed on single dataset examples, this talk presents the largest and fullest test of the method. The method was benchmarked on the Directory of Useful Decoys (DUD) datasets [4], using the same methodology described in the paper on the assessment of LASSO [5] and DOCK. For each of the DUD datasets, the known active ligands were mixed with all the decoy compounds in DUD, and the retrieval rates of INDDEx and DUD were measured when they were trained on 2, 4, and 8 of the known active ligands (Figure 2). Early retrieved compounds showed high topological differences to molecules used as training data, showing the strength of this method for scaffold hopping. This work was supported by a BBSRC case studentship with Equinox Pharma Ltd (http://www.equinoxpharma.com).

**Figure 1:**
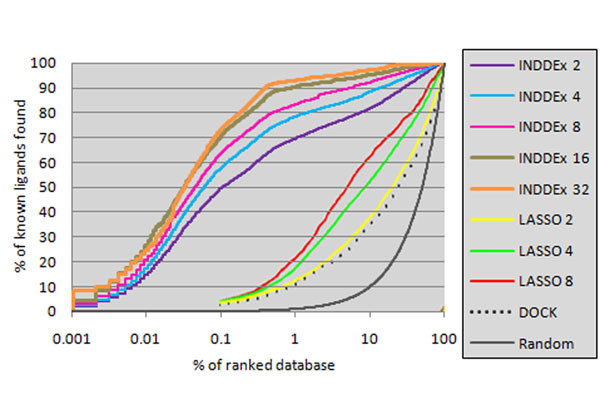
Recovery of actives in each of the DUD datasets from all decoys in the DUD, averaged across all 40 datasets.
